# The Reflectance of Human Skin in the Millimeter-Wave Band

**DOI:** 10.3390/s20051480

**Published:** 2020-03-08

**Authors:** Amani Yousef Owda, Neil Salmon, Alexander J Casson, Majdi Owda

**Affiliations:** 1 Department of Electrical and Electronic Engineering, University of Manchester, Sackville Street Building, Manchester M13 9PL, UK; alex.casson@manchester.ac.uk; 2 Department of Engineering, Manchester Metropolitan University, Chester Street, Manchester M1 5GD, UK; N.Salmon@mmu.ac.uk; 3 Department of Computing and Mathematics, Manchester Metropolitan University, Chester Street, Manchester M1 5GD, UK; m.owda@mmu.ac.uk

**Keywords:** reflectance, skin diseases, millimeter wave, radiometry, disorders

## Abstract

The millimeter-wave band is an ideal part of the electromagnetic radiation to diagnose human skin conditions because this radiation interacts only with tissue down to a depth of a millimetre or less over the band range from 30 GHz to 300 GHz. In this paper, radiometry is used as a non-contact sensor for measuring the human skin reflectance under normal and wet skin conditions. The mean reflectance of the skin of a sample of 50 healthy participants over the (80–100) GHz band was found to be ~0.615 with a standard deviation of ~0.088, and an experimental measurement uncertainty of ±0.005. The thinner skin regions of the back of the hand, the volar forearms and the inner wrist had reflectances 0.068, 0.068 and 0.062 higher than the thicker skin regions of the palm of the hand, the dorsal forearm and the outer wrist skin. Experimental measurements of human skin reflectance in a normal and a wet state on the back of the hand and the palm of the hand regions indicated that the mean differences in the reflectance before and after the application of water were ~0.078 and ~0.152, respectively. These differences were found to be statistically significant as assessed using *t*-tests (34 paired *t*-tests and six independent *t*-tests were performed to assess the significance level of the mean differences in the reflectance of the skin). Radiometric measurements in this paper show the quantitative variations in the skin reflectance between locations, sexes, and individuals. The study reveals that these variations are related to the skin thickness and water content, a capability that has the potential to allow radiometry to be used as a non-contact sensor to detect and monitor skin conditions such as eczema, psoriasis, malignancy, and burn wounds.

## 1. Introduction

The millimeter-wave band (MMW) is the electromagnetic region between the microwave and the terahertz frequency bands. The wavelength of radiation in the MMW band lies between 10 mm and 1.0 mm, covering the 30–300) GHz frequency range [1,2,3,4,5]. On account of the shorter wavelength, this band offers a higher spatial resolution measurement capability than the microwave band. Further, with a spatial resolution down to approximately half wavelength and a penetration in the skin of up to around 1.0 mm, it offers a deeper probing capability than terahertz (> 300 GHz) systems and as such it is ideal for measuring the epidermis and the dermis [6,7]. When MMW radiation strikes the skin surface, some of the radiation is reflected and the remainder is absorbed near the skin surface [7]. The amount of reflected radiation from the skin surface depends on the electrical properties of the skin layers, and in particular the relative complex permittivity of the skin that is proportional to the free water content of the skin over the MMW band [8]. An understanding of this interaction indicates the penetration depth is related to the frequency in the MMW band and this enables a particular frequency to be selected for a particular penetration depth in the skin.

Recent studies [6,8,9,10] of MMW reflectometry measured the human skin reflectance using an open-ended coaxial probe in close contact with the human body in limited numbers of participants and measurement locations. One key indicator of disease in skin is the water content, which dominates the electromagnetic properties of the skin in the microwave, millimetre and terahertz wavebands [6,11,12,13,14]. A motivation of measuring the hydration level of the skin is that it indicates the state of health of the human body and the dehydration that is counted as the most common fluid problem among aging population [15,16]. As a non-contact screening is often desirable in medical applications and since coaxial probes are unable to provide measurements over large areas without being repeatedly placed in contact with the skin, a radiometric approach seems to be a more appropriate technique for measuring the human skin reflectance under normal and wet skin conditions.

Over the MMW bands open ended coaxial probes were used for measuring the complex reflection coefficient and the relative complex permittivity of healthy and diseased skin [17,18,19,20,21,22,23,24]. Therefore, these probes have been suggested to be used as a non-invasive (in-contact technique) to distinguish between healthy skin and diseased skin. The complex reflection coefficient of unburned and burned damaged porcine skin (ex-vivo) was measured using an open-ended rectangular waveguide probe over the frequency band (30–40) GHz [17]. The measurements in [17] show that the reflectance of unburned skin is higher than that of burned damaged skin. This is due to the burning that is evaporating the water from the skin and reducing the reflectance [25,26]. However, the in vivo study in [18] that conducted on the human subject over the frequency band (30–40) GHz indicates different trends as the relative complex permittivity of the burned skin is ~10% higher than that of unburned skin. This is due to the burning that cauterizes the skin and resulting in exudates i.e., (blood and lymph fluid) around the wound site and this increases the permittivity and the reflectance of the burned skin [18]. Further, experimental measurements conducted on dry and wet skin in [19] using the open ended coaxial probe show that the relative complex permittivity of wet skin (moistened with aqueous gel) is higher than that of dry skin over the frequency band (30–100) GHz; this is due to the gel that increases the hydration level of the skin and this raises the relative complex permittivity of the wet skin. This study [19] is supported with another in vivo study [18] conducted on the palm of the hand and the back of the hand skin in a dry and wet state (skin with water) over the frequency band (30–40) GHz. The study in [18] shows that the relative complex permittivity of the wet skin is higher than that of dry skin for all measurement locations. The study conducted on healthy skin and skin with malignant lesion in [20] indicates significant differences in the dielectric permittivity between healthy skin (65% and 75% water content) and malignant lesion tissue (81.6% water content). These differences are due to the different water contents of healthy skin and malignant tissue [7]. The relative complex permittivity of healthy skin and skin with basal cell carcinoma (BCC) over the (100–300) GHz frequency band [21,22] indicate that the relative complex permittivity of the skin with BCC is higher than that of healthy skin as the water content of the skin with BCC is higher than that of healthy skin [27]. Further, the in vivo study conducted in [23] shows a well-defined contrast the relative complex permittivity of healthy skin and skin with BCC at 42 GHz. In addition, [24] proposed the near field probe to distinguish between healthy skin and skin with BCC. The results obtained from these studies [17,18,19,20,21,22,23,24] suggested that open ended coaxial probes can be used over the MMW bands for early detection of skin diseases.

The use of the open ended coaxial probe over the MMW band required that the probe should be in direct contact with the human body and this involves exposing the human body to man-made radiations from the vector network analyser. Further, the use of the open-ended coaxial probe required constant pressure to be applied on the measured site as the varying size of the air gap between the measured site and the probe affects the measured relative complex permittivity [28]. This makes the open ended coaxial probe not feasible to be used in such situations where we cannot put the probe in direct contact with the skin under constant pressure. As an alternative; radiometry as a non-contact passive sensor is suggested to be used in this paper for measuring the human skin reflectance and possibly for a non-invasive diagnosis of skin diseases and disorders.

**Figure 1:**
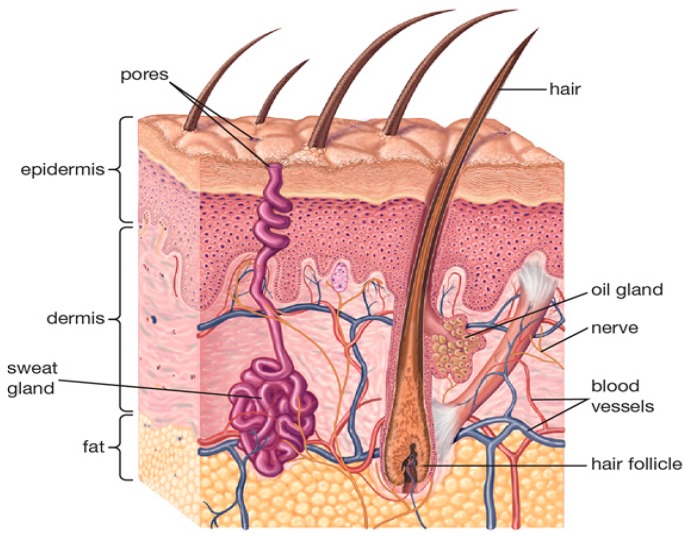
Schematic for human skin structure [38]. Radiation in the lower frequency portion of the MMW band penetrates down into the dermis. The schematic obtained from [38] an open access encyclopædia (Encyclopædia Britannica).

Human skin has a layered structure comprising the epidermis (outer layer), the dermis (inner layer), and the hypodermis (subcutaneous fat layer), as illustrated in Figure 1. For adults, skin covers a total surface area of ~1.7 m^2^ and constitutes ~15% of the total body weight [29,30]. Skin thickness varies with location over the body, and sex of the individual [31]. Many studies reveal strong correlation between skin dielectric properties, skin thickness, water content and hydration level, all of which vary with sex and location on the body [31,32,33,34,35,36]. Males generally were found to have thicker skin than females for all age groups [34,35,37], whereas the skin of females was found to have slightly higher hydration levels compared with that of males [33].

Although, human skin is the largest multifunctional organ in the human body, it provides a physical barrier against the penetration of viruses, bacteria, and inorganic matter [29,30,34]. To our knowledge no detailed investigations of a statistical nature have been carried out on the reflectance of the human skin. This study redresses this balance by analysing the reflectance of the skin for a sample of 50 healthy participants at different locations of the human arm, for subjects of different sexes and for the skin in a wet and normal state, over the 80 GHz to 100 GHz region of the millimeter wave band. These results will provide foundational measurements to support potential medical applications (non-invasive diagnosis of skin diseases). In our previous research [7,25,39,40,41,42] we have investigated the signature i.e., (emissivity) of the human skin over the MMW band for security screening (anomalies detection) and medical applications (monitoring wound healing under dressing materials) using different samples of healthy participants and porcine skin. These works, and [39] in particular, focused on measuring the emissivity of the skin and no statistical comparisons were performed. This paper reports reflectivity measurements from a new sample of 50 healthy participants, allowing the significant differences in reflectance between males and females, between different sites on the forearm and hand, and between wetted and dry skin to be reported for the first time. While emissivity and reflectivity can be closely related mathematically (as discussed below), there remains a gap in the literature explicitly measuring reflectivity and demonstrating statistically significant differences in it from different parts of the body. The result is a new methodology for measuring the human skin reflectance in non-contact with the human body using radiometry (passive sensor) for use in a wide range of remote sensing applications. Our findings suggest that radiometry can be used as a non-contact sensor for measuring the human skin reflectance and potentially for a non-invasive diagnosis of diseased skin, where the disease affects the hydration level of the skin or the skin thickness, as in the case with eczema, malignancy, dehydration, burn wound, and psoriasis.

The remainder of the paper is structured as follows: Section 2 describes the experimental methodology for measuring the human skin reflectance, Section 3 presents the experimental results and the statistical analysis on the data, Section 4 discusses the results and highlights motivations for future directions, and Section 5 draws the overall conclusions of the paper.

## 2. Materials and Methods

### 2.1. Participants

Fifty healthy participants (30 males and 20 females) with no history of skin disease were recruited in this study. The participants had a mean and a standard deviation (± SD) in their age: 30.2 ± 4.25 years, mass: 70.5 ± 10.95 kg, and height: 1.62 ± 0.092 m. The study was approved by the ethics committee of Manchester Metropolitan University under ethics reference no: SE151630CA1. A written consent form was obtained from all participants before performing the measurements.

### 2.2. Measurement Locations

**Figure 2:**
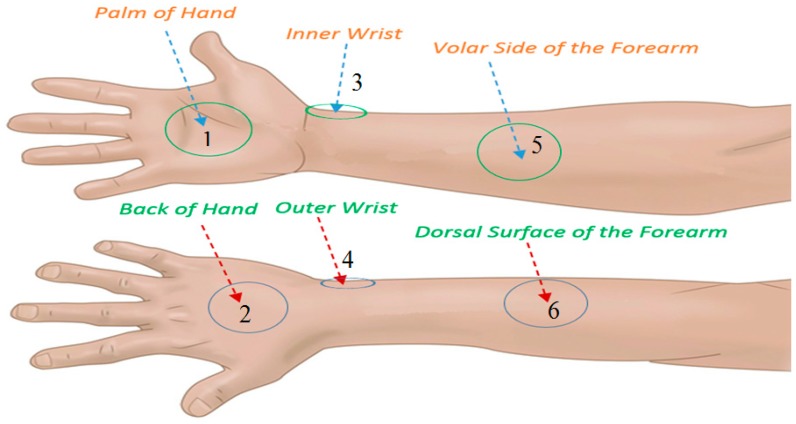
Six regions on the arm where the reflectance of the skin is measured.

The reflectance measurements for normal healthy skin were made at six locations on the arm and these were: (1) the palm of the hand, (2) the back of the hand, (3) the inner wrist, (4) the outer wrist, (5) the volar side of the forearm (50 mm from the inner elbow), and (6) the dorsal surface of the forearm (50 mm from the outer elbow) as illustrated in Figure 2. These locations were chosen since the water content and the skin thickness are markedly different [43] and the measurements were repeated 5 times to obtain a mean value.

### 2.3. Experimental Setup and Calibration

**Figure 3:**
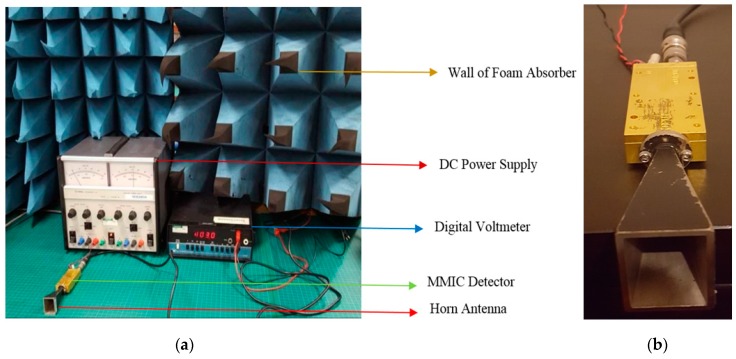
(**a**) Experimental setup showing the horn antenna and the MMIC detector. The detector was connected to a digital voltmeter and a DC power supply. A wall of anechoic chamber is surrounding the majority of the system. (**b**) The radiometer.

A radiometer (direct detection model, made by Millitech, Northampton, MA 01060, US), effective over the frequency band 80 GHz to 100 GHz, was utilised for measuring the reflectance of the human skin. The equipment for measurement consisted of a horn antenna operating over the band from 80 GHz to 100 GHz. The antenna was connected to the millimeter-wave monolithic integrated circuit (MMIC) detector. The detector output was connected to a digital voltmeter and to a DC power supply [7,25], as shown in Figure 3.

The W-band horn antenna (model number: AS4341, Atlan TecRF, Essex, UK) had a rectangular aperture (30 mm × 25 mm) and a nominal gain of 20 dBi. During the experiment, the horn antenna was fixed to measure emission from the subject and from the hot and cold calibration sources. It typically took about 1.0 min to complete this measurement process per site, allowing for a settling time, which minimises systematic errors associated with drift.

The MMIC detector consisted of zero bias diode detectors, a two-stage low-noise amplifier (LNA), and a buffer amplifier. The complete system, except for an opening for the subject to be measured, was enclosed in an anechoic region (where there is no radiometric emission from people or lower emissions from outdoors) made by surrounding the detector and the horn antenna with carbon loaded absorbing foam. Radiometers have the performance metric of noise temperature measured in Kelvin; the lower the figure the more sensitive the system. The noise temperature for the radiometer was measured to be 453.7 K, which represents a good performance for measuring the human skin reflectance.

Two pieces of carbon loaded foam absorber (model: Eccosorb AN-73, Laird, Shanghai, China) acted as cold and hot load calibration sources; the absorbers had a rectangular shape and dimensions (length = 170 mm, width = 150 mm, and thickness 10 mm). These dimensions were chosen to fill the beam pattern of the horn antenna and to reduce the systematic uncertainty during the calibration. The carbon loaded foam absorbers have measured emissivity values greater than 0.99 over the frequency band 80–100 GHz, thus they behave as good approximations to a black body radiator.

A standard thermocouple (model number: L812, Leaton, Suffolk, UK) and an infrared thermometer (model number: N85FR, Maplin, Manchester, UK) were used to measure the temperatures of the skin and the calibration sources. The results obtained from the two thermometers were the same with a relative measurement uncertainty of less than 0.1 °C. Further, as the infrared thermometer could measure the temperature of the skin in a non-contact way, it was chosen to measure the skin temperature.

The amount of radiometer self-emission reflected back from subjects was investigated by moving a metal plate in a distance 1.0 cm from the horn antenna beam. The mean level of self-emission reflected back from the metal plate (which has a reflectance of 100% in the millimeter wave band) was measured to be in the range of 294–295 K. This indicates that the radiation temperature from the metal plate is approximately the same as the ambient temperature. This indicated no parasitic signals associated with the self-emission were present, which might have otherwise corrupted measurements.

### 2.4. Reflectance Measurements

**Figure 4:**
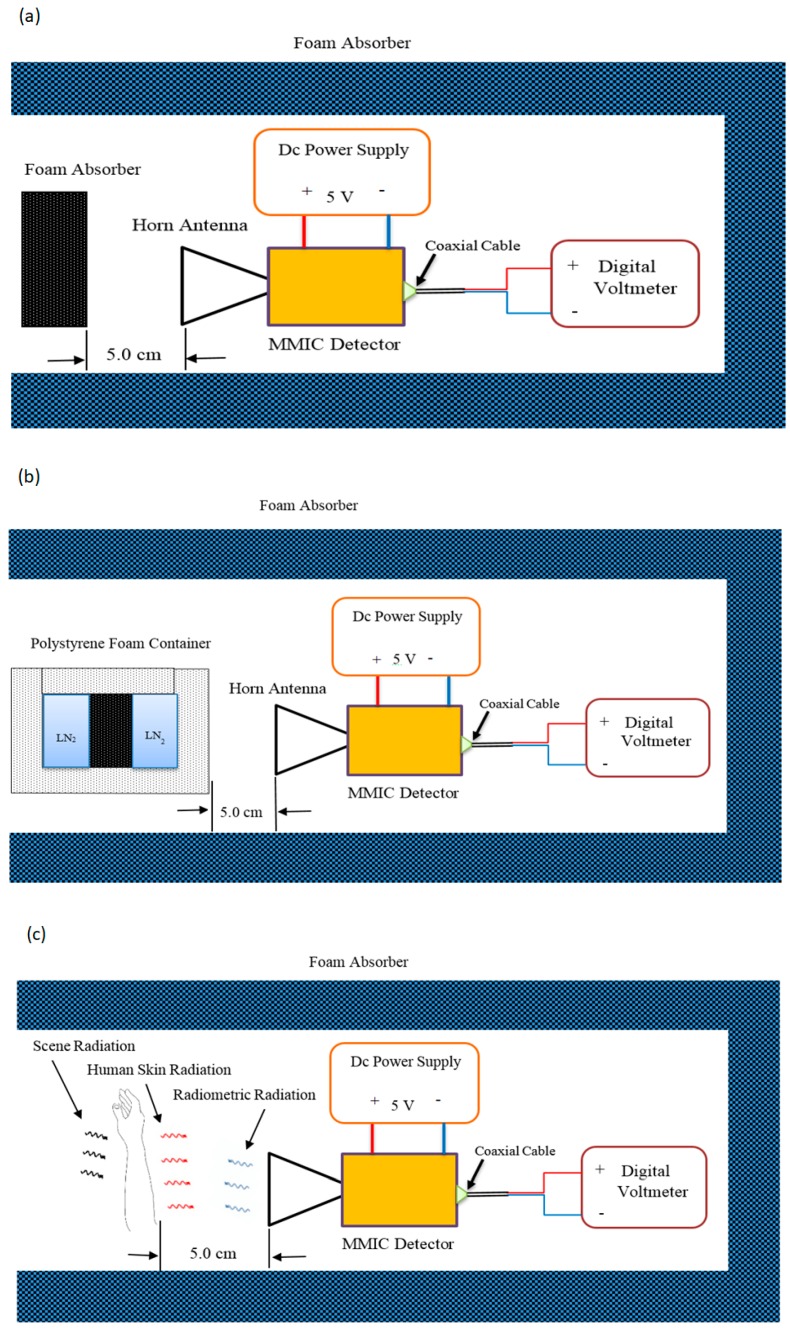
Schematics of the experimental setup inside the walls (blue-grey) of the anechoic chamber, showing the hot (**a**) and the cold (**b**) calibration procedures and the measurement of the skin (**c**).

The horn antenna was located at a distance (5.0 cm) from three different radiation sources and these were: (1) ambient temperature source calibration (hot load) – a piece of carbon loaded foam absorber with T_H_ = T_ambient_ = 295 K; (2) liquid nitrogen (LN_2_) source calibration (cold load) – a piece of carbon loaded foam absorber was dipped in the liquid nitrogen bucket with T_C_ = 79 K; and (3) the participant’s area of the skin to be measured as illustrated in Figure 4. The distance 5.0 cm was chosen to minimize the chances of subjects accidentally touching and moving the measurement apparatus. A greater distance between the measured subject and the horn antenna would lead to measurements having poorer spatial resolution. The system response was assumed to be linear, since the measurements were performed indoors in an anechoic environment. The output of the receiver in Volts for an ambient temperature source calibration can be expressed as [39,41,44]:

(1) $$V_{H}=\alpha \left(T_{H}+T_{N}\right),$$

where, $T_{H}$ is the hot load temperature measured in Kelvin, α is the receiver responsivity measured in Volts per Kelvin, and $T_{N}$ is the receiver noise temperature in Kelvin. For the liquid nitrogen source calibration, the output of the receiver is [39,41,44]:

(2) $$V_{C}=\alpha \left(T_{C}+T_{N}\right)$$

Subtraction of (1) and (2) provides the receiver responsivity, α:

(3) $$\alpha =\frac{\left(V_{H}-V_{C}\right)}{\left(T_{H}-T_{C}\right)}$$

The radiation temperature of the skin can be expressed in terms of the skin emissivity η, the skin temperature $T_{s}$, and the background illumination temperature $T_{o}$ [45] as:

(4) $$T_{b}=\left(1-\eta \right)T_{o}+T_{s}\eta$$

From (1) to (4), and equating $T_{o}$ to $T_{H}$, since the background illumination temperature is the same as ambient temperature in an anechoic environment, the measured emissivity of the human skin can be expressed as [39,41]:

(5) $$\eta =\frac{\left(V_{s}-V_{H}\right)\left(T_{H}-T_{C}\right)}{\;\left(T_{s}-T_{H}\right)\left(V_{H}-V_{C}\right)}$$

Conservation of electromagnetic energy provides a relationship between the emissivity η, the reflectance R, and the transmittance T of the skin surface as [42,46]:

(6) 1=R+T+η

As the human skin is opaque (T=0) over the MMW band from 80 GHz to 100 GHz [7], the reflectance of the skin can be expressed as:

(7) R=1−η

By substituting (5) in (7), the reflectance of the skin is:

(8) $$R=\frac{T_{S}\left(V_{H}-V_{C}\right)+T_{H}\left(V_{C}-V_{S}\right)+T_{C}\left(V_{S}-V_{H}\right)}{\left(T_{s}-T_{H}\right)\left(V_{H}-V_{C}\right)}$$

An infrared thermometer was used to measure the temperatures of the human skin, $T_{S}$ directly before and after the measurements. A digital voltmeter was used to measure the output voltage for the target area of the skin, $V_{S}$. All measurements were obtained at an ambient temperature of ~295 K and the voltage measurements were up to 100 mV with a precision of 0.1 mV. Error propagation through (8) indicates that the uncertainty on the measured reflectance is ±0.005.

### 2.5. Reflectance Measurements for Wet Skin

The water content of the skin dominates its electromagnetic behavior in the millimeter wave band [6,11,12,13,14], so a measurement was made to quantify this statement at 90 GHz. Firstly reflectance measurements of normal clean skin on the back and the palm of the hand were made. Then these areas of the skin were covered with (non-ionising) water, which was then left to be absorbed for 2–4 min. After such time, when there was no water left visible on the skin, second measurements of the reflectance were made. This methodology was applied on a new sample of 12 healthy participants (6 females and 6 males) and the measurements were repeated 5 times to obtain a mean value.

### 2.6. Assessing the Significance Level of the Mean Differences in the Reflectance

The significance level of the mean differences in the reflectance values between different locations on the arm and subject to the same sex was assessed using the paired *t*-test, whereas the independent *t*-test was used to assess the significance level of the mean differences in the reflectance values between males and females at similar measurement locations. In both cases if the *p*-value is lower than the critical significance level of 0.05; this means that the mean differences in the reflectance values are significant. Otherwise, differences are insignificant.

## 3. Results

This section presents reflectance measurements made on human skin over the frequency band 80 GHz to 100 GHz. The measurements were conducted on a sample of 50 healthy participants (20 females and 30 males) at six locations on the arm. Then reflectance measurements were performed on the skin under normal and wet skin conditions on a different sample of 12 healthy participants (6 females and 6 males).

### 3.1. Reflectance Measurements for the Whole Sample

The mean, the standard deviation (SD), and the standard error in the mean (SEM = σ/√n; where n is the sample size) of the human skin reflectance for a sample of 50 healthy participants at all measurement locations are summarised in Table 1.

The experimental results in Table 1 indicate that the mean differences in the reflectance values between the back and the palm of the hand, the volar and the dorsal surface of the forearm and the inner and the outer wrist regions are: 0.068, 0.068 and 0.062 with a sample standard deviation in the differences of 0.0396, 0.0396 and 0.0375, respectively. These differences are due to the varying skin thicknesses and the water contents, both of these being dependent on the location on the body and they are different between individuals [32,33,34]. The thinner skin regions with blood vessels closed to the skin surface [43,47] makes the skin more reflective and these results in higher reflectance in the back of the hand, the volar side and the inner wrist regions.

### 3.2. Reflectance Measurements for Female Participants

**Figure 5:**
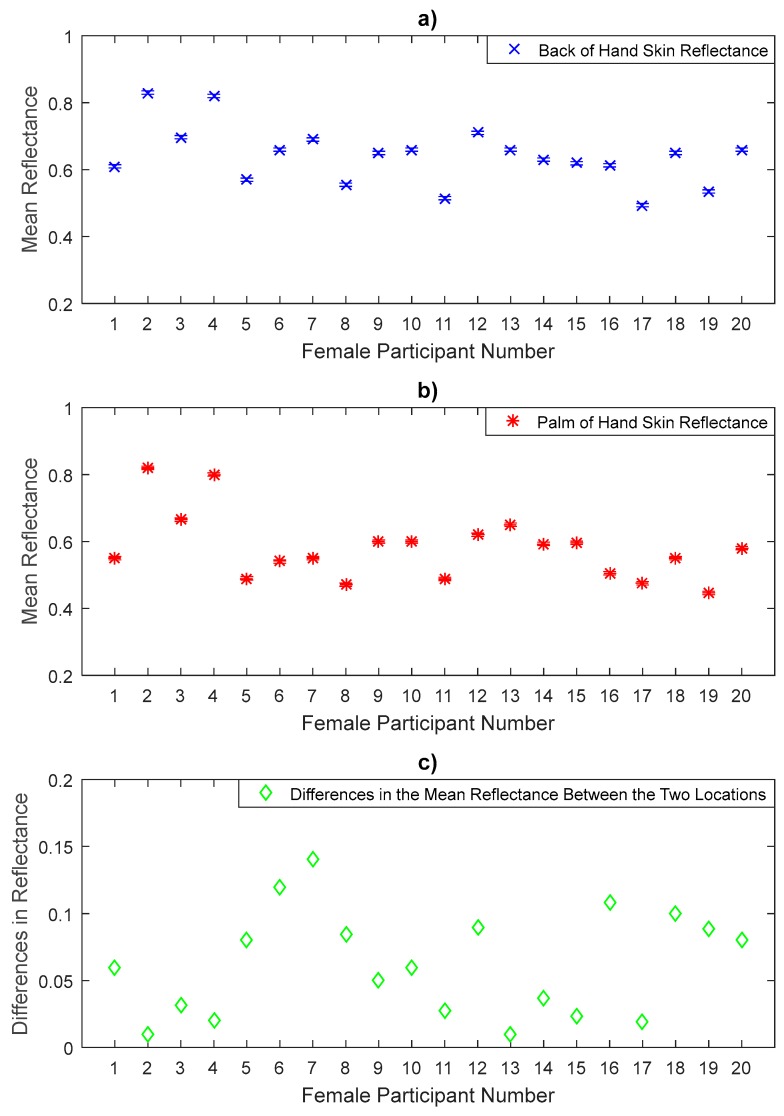
Measurements of human skin reflectance on the back of hand (**a**) and the palm of hand skin (**b**) for a sample of 20 female participants. The mean differences in reflectance values between the two locations (**c**) are in the range of 0.01 to 0.14.

**Figure 6:**
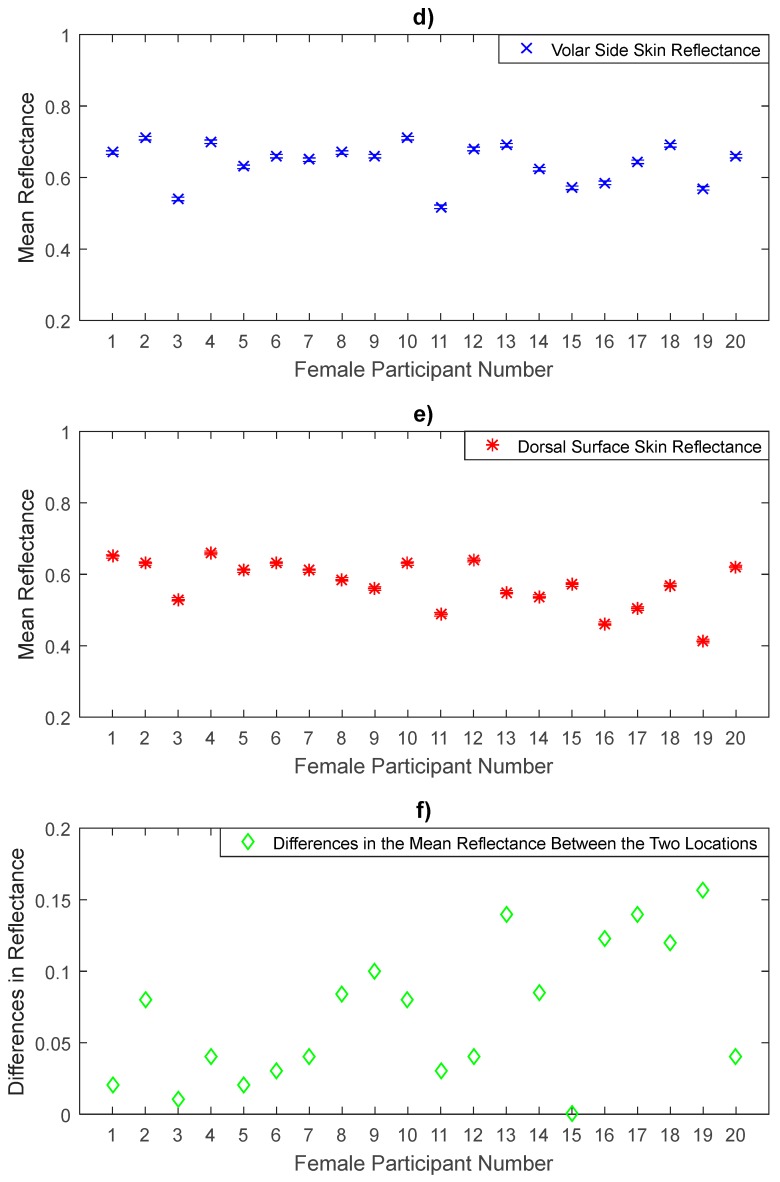
Measurements of human skin reflectance on the volar side (**d**) and the dorsal surface skin (**e**) for a sample of 20 female participants. The mean differences in reflectance values between the two locations (**f**) are in the range of 0 to 0.157.

**Figure 7:**
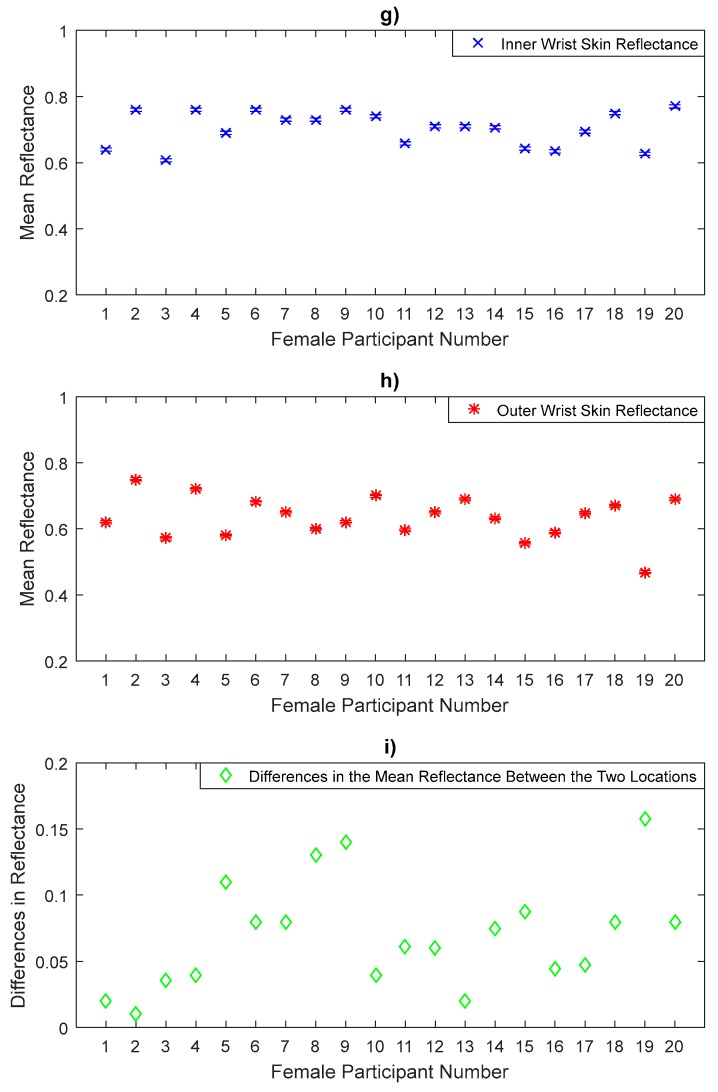
Measurements of human skin reflectance on the inner wrist (**g**) and the outer wrist (**h**) for a sample of 20 female participants. The mean differences in reflectance values between the two locations (**i**) are in the range of 0.01 to 0.158.

The measurements in Figure 5, Figure 6 and Figure 7 represent the mean values of the reflectance for a sample of 20 female participants, with error bars representing the systematic uncertainty of ±0.005. The measurements show significant variations in the reflectance between individuals and locations on the arm. The measurements indicate that females have a sample mean reflectance of 0.629 with a sample standard deviation of 0.085, generating a standard error in the mean of 0.0189.

The measurements in Figure 5 show that the reflectance for the back of the hand skin is higher than that of the palm of the hand skin for all female participants. Statistical analysis on the data of a sample of 20 female participants indicates that the back of the hand skin has a mean reflectance of 0.641 with a standard deviation of 0.0846, this generates a standard error in the mean of 0.0189, whereas the palm of the hand skin has a mean reflectance of 0.579 with a sample standard deviation of 0.097, and a standard error in the mean of 0.0216. The sample means of the reflectance values of the back of the hand skin are higher by 0.062 than that of the palm of the hand. These differences are significant as the *p*-value (1.0 × 10^−6^) obtained from the paired *t*-test is lower than the critical significance level of 0.05. This indicates that there are significant differences in the mean reflectance values between the thinner skin region of the back of the hand and the thicker skin region of the palm of the hand [43,47]. Note that as an exploratory study the *p*-value presented herein is uncorrected for multiple comparisons and results should be interpreted being aware of this.

Experimental measurements of the volar side and the dorsal surface skin reflectance in Figure 6 show that the volar side skin reflectance is higher than that of the dorsal surface skin for all female participants. Statistical analysis on the data indicates that the volar side skin has a mean reflectance of 0.642 with standard deviation of 0.0552, generating a standard error in the mean of 0.0124, whereas the dorsal surface skin has a mean reflectance of 0.573 with a sample standard deviation of 0.0658, generating a standard error in the mean of 0.0147. The sample means of the reflectance values of the volar side skin are higher by 0.069 than that of the dorsal surface. These differences were found to be significant as the uncorrected *p*-value (4.0 × 10^−6^) obtained from the paired *t*-test was lower than the critical significance level of 0.05.

The reflectance measurements of the inner wrist and the outer wrist regions in Figure 7 show that the inner wrist reflectance is higher than that of the outer wrist skin for all female participants. Statistical analysis on the data indicates that the inner wrist skin has a mean reflectance of 0.704 with a standard deviation of 0.0511, generating a standard error in the mean of 0.011, whereas the outer wrist skin has a mean reflectance of 0.634 with a sample standard deviation of 0.0635, generating a standard error in the mean of 0.014. The sample means of the reflectance values of the inner wrist skin are higher by 0.07 than the outer wrist skin. These differences were found to be significant as the uncorrected *p*-value (3.1 × 10^−7^) obtained from the paired *t*-test was lower than the critical significance level of 0.05.

### 3.3. Reflectance Measurements for Male Participants

**Figure 8:**
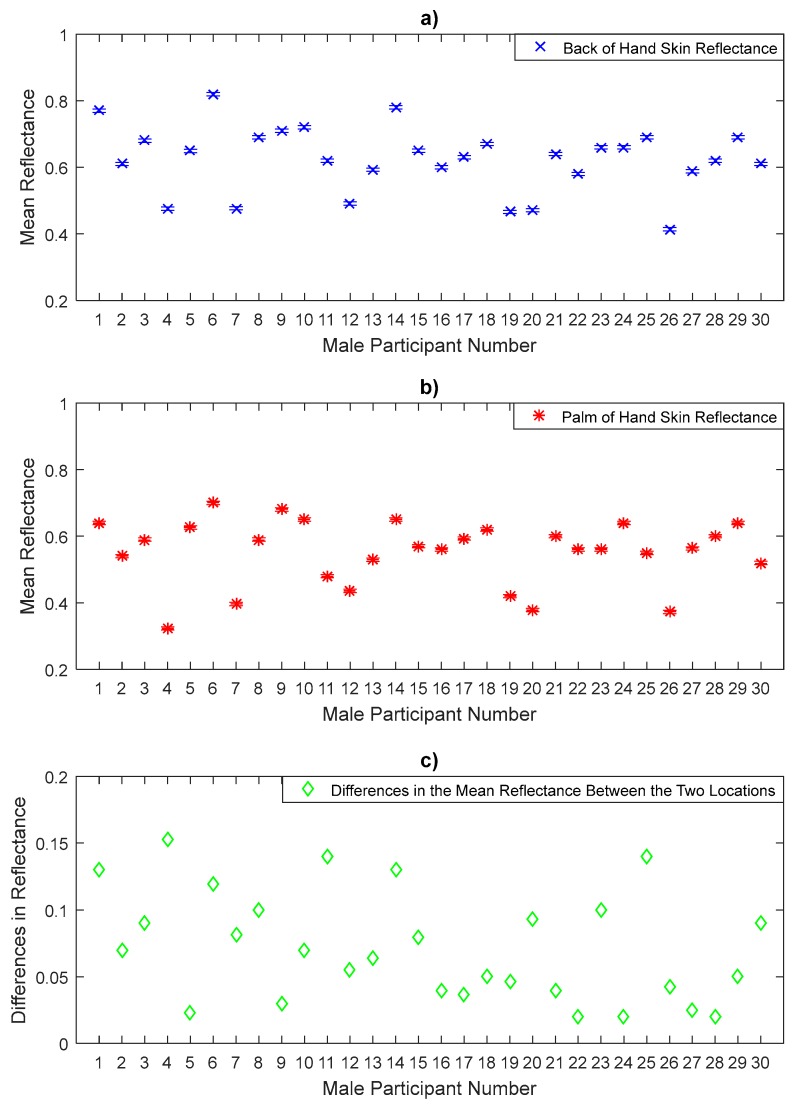
Measurements of human skin reflectance on the back of hand (**a**) and the palm of hand skin (**b**) for a sample of 30 male participants. The mean differences in reflectance values between the two locations (**c**) are in the range of 0.02 to 0.153.

**Figure 9:**
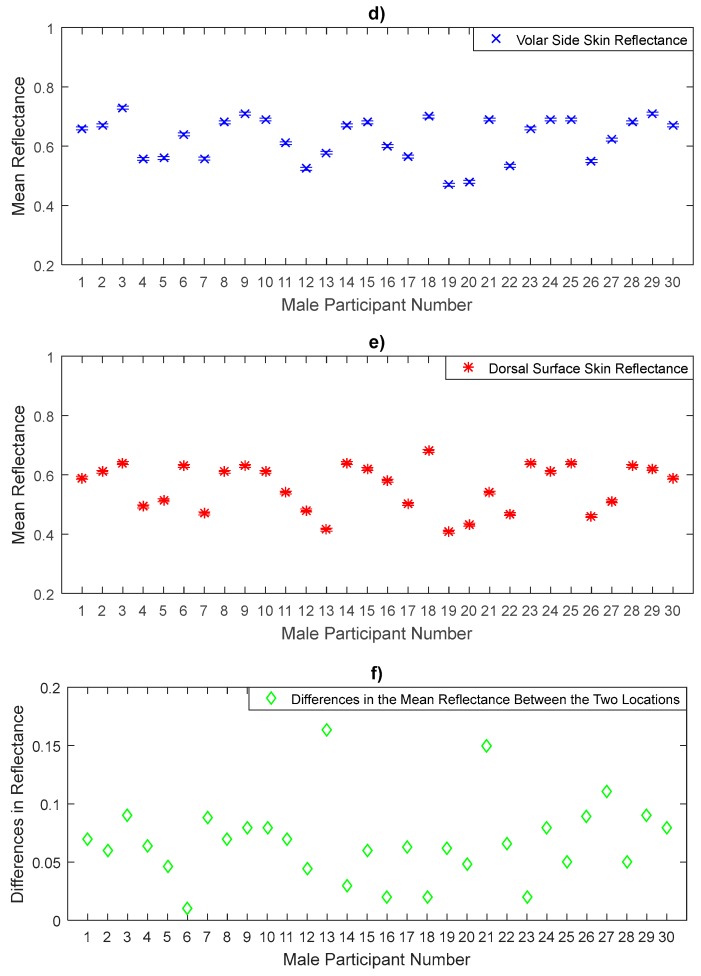
Measurements of human skin reflectance on the volar side (**d**) and the dorsal surface skin (**e**) for a sample of 30 male participants. The mean differences in reflectance values between the two locations (**f**) are in the range of 0.01 to 0.163.

**Figure 10:**
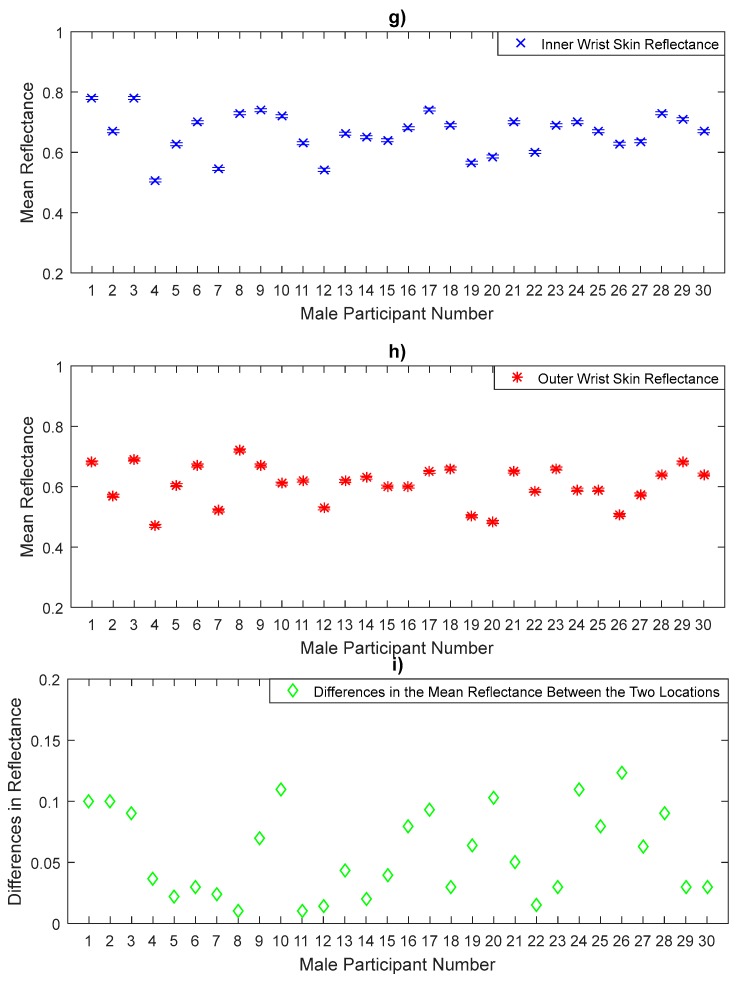
Measurements of human skin reflectance on the inner wrist (**g**) and the outer wrist (**h**) for a sample of 30 male participants. The mean differences in reflectance values between the two locations (**i**) are in the range of 0.01 to 0.123.

The measurements in Figure 8, Figure 9 and Figure 10 represent the mean values of the reflectance for a sample of 30 male participants. The measurements indicate that males have a sample mean reflectance of 0.606 with a sample standard deviation of 0.089, generating a standard error in the mean of 0.016. Reflectance measurements for males show a similar trend to that of females in terms of the differences in the mean reflectance values between the thinner skin region (back of hand, volar side of the forearm, and inner wrist) and the thicker skin region (palm of hand, dorsal surface of forearm, and outer wrist). The results obtained from the paired *t*-tests for male participants indicate significant differences in the mean reflectance values between the back and the palm of the hand (*p*-value = 1.6 × 10^−10^), the volar and the dorsal surface of the forearm (*p*-value = 1.2 × 10^−11^) and the inner and the outer wrist regions (*p*-value = 1.2 × 10^−9^).

The measurements in Figure 8 show that the reflectance for the back of the hand skin is higher than that of the palm of the hand skin for all male participants. Statistical analysis on the data of a sample of 30 male participants indicates that the back of the hand skin has a mean reflectance of 0.624 with a standard deviation of 0.097, this generates a standard error in the mean of 0.0177, whereas the palm of the hand skin has a mean reflectance of 0.553 with a sample standard deviation of 0.096, and a standard error in the mean of 0.0176. The sample means of the reflectance values of the back of the hand skin are higher by 0.071 than that of the palm of the hand.

Experimental measurements of the volar side and the dorsal surface skin reflectance in Figure 9 show that the volar side skin reflectance is higher than that of the dorsal surface skin for all male participants. Statistical analysis on the data indicates that the volar side skin has a mean reflectance of 0.628 with standard deviation of 0.072, generating a standard error in the mean of 0.013, whereas the dorsal surface skin has a mean reflectance of 0.56 with a sample standard deviation of 0.078, generating a standard error in the mean of 0.014. The sample means of the reflectance values of the volar side skin are higher by 0.068 than that of the dorsal surface.

The reflectance measurements of the inner wrist and the outer wrist regions in Figure 10 show that the inner wrist reflectance is higher than that of the outer wrist skin for all male participants. Statistical analysis on the data indicates that the inner wrist skin has a mean reflectance of 0.664 with a standard deviation of 0.068, generating a standard error in the mean of 0.012, whereas the outer wrist skin has a mean reflectance of 0.607 with a sample standard deviation of 0.064, generating a standard error in the mean of 0.012. The sample means of the reflectance values of the inner wrist skin are higher by 0.057 than the outer wrist skin.

### 3.4. Comparison in Skin Reflectance between Female and Male Participants

Experimental measurements of the skin reflectance at a center frequency of 90 GHz from a sample of 50 healthy participants indicate that there is a scatter in the mean reflectance values over a range from 0.323 to 0.83. Calculating the sample mean reflectance values for the 20 females and 30 males separately over all measurement locations indicates that the sample mean of the female reflectance is higher by ~0.023 than that of male reflectance. This difference is due to the skin of males being thicker than that of females in all ages [34,35]. The thicker skin means that the blood vessels are further from the skin surface, and the absorption in the thicker skin will reduce the reflectance. Alternatively, thinner skin, with higher reflecting blood vessels closer to the surface, will increase the reflectance of the skin. Table 2 shows an overview of the statistical analysis of the human skin reflectance for a sample of 20 females and 30 males separately over all measurement locations.

Table 2 indicates differences in the mean reflectance values between female and male groups. The significance level of these differences was assessed using the independent *t*-test as illustrated in Table 3.

### 3.5. Skin Reflectance for Female Participants Under Normal and Wet Skin Conditions

**Figure 11:**
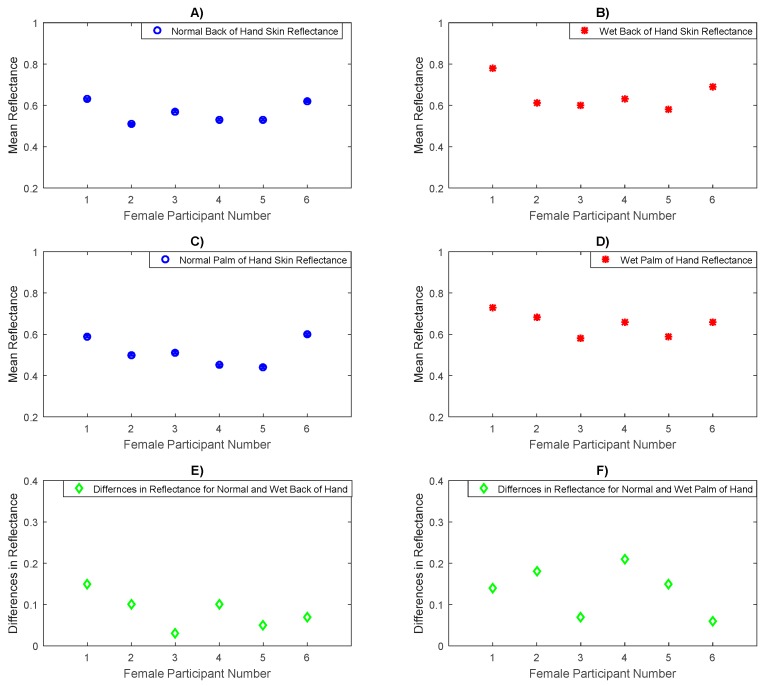
Measurements of human skin reflectance on normal and wet back of hand and palm of hand skin for a sample of 6 female participants. The mean differences in reflectance between the normal (**A**) and the wet back of hand skin (**B**) are in the range of 0.03 to 0.15 as illustrated in (**E**) whereas the mean differences in reflectance between the normal (**C**) and the wet palm of hand skin (**D**) are in the range of 0.06 to 0.21 as illustrated in (**F**).

Experimental measurements for a sample of six female participants in Figure 11, indicate that the mean reflectance values for the back of the hand skin and the palm of the hand skin after moistening with water is significantly higher than the mean reflectance of the skin in normal state (before moistening with water) for the two measurement locations.

Statistical analysis on the data indicates that the mean difference in reflectance for the back of the hand skin before and after moistening with water is ~0.0833 with a sample standard deviation in the differences of ~0.039, whereas the mean difference in the reflectance for the palm of the hand skin before and after moistening with water is ~0.135 with a standard deviation in the differences of ~0.054. These differences are due to the applied water, which increases the hydration level of the skin [8] and this makes the reflectance of the skin higher. The results obtained from the paired *t*-tests indicate significant differences in the mean reflectance values between the normal and the wet back of hand skin (*p*-value = 4.9 × 10^−3^) and the normal and the wet palm of the hand skin (*p*-value = 2.6 × 10^−3^).

### 3.6. Skin Reflectance for Male Participants Under Normal and Wet Skin Conditions

**Figure 12:**
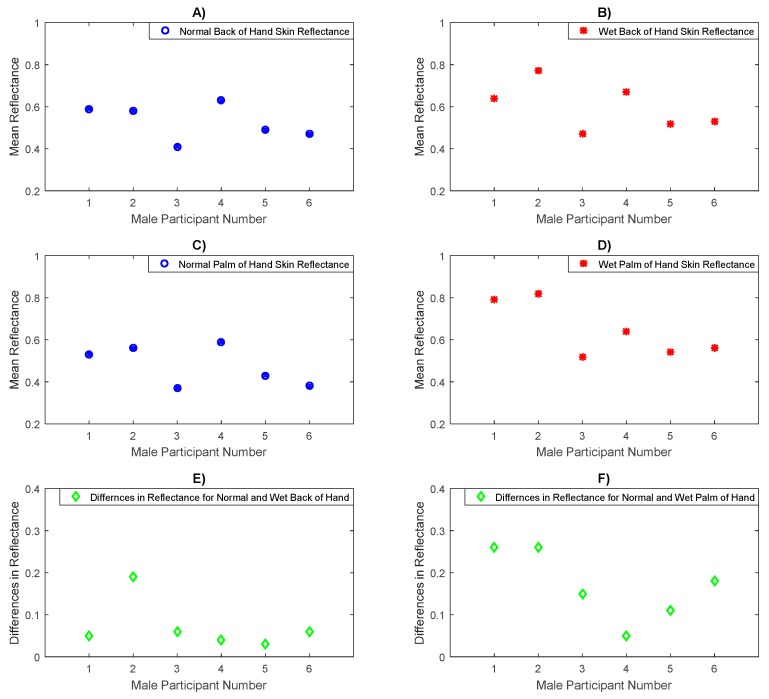
Measurements of human skin reflectance on normal and wet back of hand and palm of hand skin for a sample of 6 male participants. The mean differences in reflectance between the normal (**A**) and the wet back of hand skin (**B**) are in the range of 0.03 to 0.19 as illustrated in (**E**) whereas the mean differences in reflectance between the normal (**C**) and the wet palm of hand skin (**D**) are in the range of 0.05 to 0.26 as illustrated in (**F**).

Experimental measurements for a sample of six male participants in Figure 12 show that the mean reflectance for the back of the hand skin and the palm of the hand skin after moistening with water is higher than that of the back of the hand and the palm of the hand skin before adding water by mean values of 0.072 and 0.168 and standard deviations of 0.054 and 0.076 respectively. The differences between the wet and the normal palm of hand skin are higher than that of the back of the hand skin for both sexes, and this is due to thick stratum corneum (SC) layer that can retain water [8] and make the hydration level for the palm of the hand skin substantially higher in a wet state compared with a normal state. The results obtained from the paired *t*-tests indicate significant differences in the mean reflectance values between the normal and the wet back of hand skin (*p*-value =3.1 × 10^−2^) and the normal and the wet palm of the hand skin (*p*-value = 4.2 × 10^−3^).

## 4. Discussion

This paper presents measurements of the human skin reflectance from a sample of 50 healthy participants over the frequency band 80–100 GHz. Estimating the sample mean reflectance values for the 30 males and 20 females separately indicates that there are differences in the mean reflectance values between males and females in all measurement locations. These differences were found to be insignificant in the back of the hand, the palm of the hand, the volar side, the dorsal surface, and the outer region, whereas it is significant in the inner wrist region as assessed using the uncorrected *p*-value of the independent *t*-test.

The measurements over a sample of 50 participants in Figure 5, Figure 6, Figure 7, Figure 8, Figure 9 and Figure 10 indicate that human skin reflectance varies from person to person for both sexes. These variations are due to the skin thickness, water content and blood circulation, these factors having some dependency on weather conditions, the time of day, the age, the sex, and the state of health of the participants [33,35].

Experimental measurements of a sample of 20 female participants in Figure 5, Figure 6 and Figure 7 indicate that there are differences in the mean reflectance values over all measurement locations. The significance level of these differences is assessed using the paired *t*-tests as summarised in Table 4. These differences are due to the skin thickness and blood vessels. The thinner skin regions make the blood vessels closer to the skin surface and this increases the reflectance of the human skin significantly, whereas the thicker skin regions make the blood vessels further from the skin surface and this makes the reflectance of the skin lower than that of the thinner skin regions.

Similarly, the significance level of the mean differences in the reflectance values between the six measurement locations on the arm over a sample of 30 male participants in Figure 8, Figure 9 and Figure 10 is assessed using the paired *t*-test as summarised in Table 5.

Experiment measurements for a sample of six female participants before and after moistening with water the back of the hand, and the palm of the hand skin, in Figure 11 indicate that the mean difference in the reflectance for the back of the hand skin before and after adding water is ~0.0833 with a sample standard deviation in the differences of ~0.039, whereas the mean difference in the reflectance for the palm of the hand skin before and after adding water is ~0.135 with a sample standard deviation in the differences of ~0.054. These differences are statistically significant (as assessed using the paired *t*-test) and they confirm a strong correlation between the human skin reflectance and the hydration level of the skin.

Experiment measurements of the reflectance for a sample of six male participants before and after adding water on the back of the hand, and the palm of the hand skin, in Figure 12 indicate that the mean difference in the reflectance for the back of the hand skin before and after adding water is ~0.072 with a sample standard deviation in the differences of ~0.054, whereas the mean difference in the reflectance for the palm of the hand skin before and after adding water is ~0.168 with a sample standard deviation in the differences of ~0.076. These differences are statistically significant as assessed using the uncorrected *p*-value of the paired *t*-test.

The measurements presented in this paper show a strong correlation between the skin reflectance, thickness and the water content. As an imaging sensor, radiometry can deliver spatial resolutions down to around half of the wavelength of the radiation used, which can be ~1.0 mm for the millimetre wave band. This property enables highly localized, non-contact measurements to be made just below the skin surface. This indicates that radiometry could be used as a non-contact sensor to detect and monitor skin disease or damage, where the disease or the damage alters the water content or the skin thickness such as dehydration, eczema, malignancy, psoriasis and burn wounds.

As a plan for future work, it is recommended that measurements be made on patients having different types of skin diseases such as burn, psoriasis, malignancy, and eczema. The mean reflectance values from people with different skin diseases/conditions are needed to be measured and identified and then compared with the mean reflectance values of healthy skin subject to the same region obtained from big population sample. This involves considering the standard deviation of healthy population so any deviations from the standard norms should be identified as well as unusually high or low levels of the mean reflectance values. In general; the lower reflectance values of the skin are indicative of a dry skin conditions, whereas the higher reflectance values are indicative of the presence of exudates, infection, malignancy, a non-healing state of burn wounds.

## 5. Conclusions

Experimental measurements of human skin reflectance of a sample of 50 healthy participants (20 females and 30 males) over the frequency band 80–100 GHz show that the mean reflectance of male skin is lower than that of female by ~0.023 with an experimental measurements uncertainty of ±0.005. This supports the knowledge that the skin of male is thicker than that of female in all ages. Reflectance measurements also show that the reflectance of thicker layers of skin in the human body, such as the palm of the hand, the dorsal surface of the forearm, and the outer wrist skin is lower than those of the back of the hand, the volar side of the forearm, and the inner wrist by ~0.068, ~0.068, and ~0.062 respectively. This indicates a significant difference in the reflectance of the skin between the thinner and the thicker skin regions as assessed using the *t*-tests; the higher reflectance is indicative of the thinner skin whereas the lower reflectance is inductive of the thicker skin.

Experimental measurements of human skin reflectance in a normal and a wet state on the back of the hand and the palm of the hand regions for a sample of 12 participants (six females and six males) indicate that the mean differences in reflectance before and after the application of water is ~0.078 and ~0.152 respectively. These differences confirm a strong correlation between the human skin reflectance and the hydration level of the skin.

Research continues in this area to identify the mean reflectance values for all regions of the human body and for all classes (males, females, ages, ethnicities) of healthy individuals. Mean reflectance values from people with different skin conditions are needed to be measured and compared with these standard norms and where differences are recorded; action can be taken to determine the underlying causes. In a similar way, the time histories of the mean reflectance values of people with different skin conditions are needed to be monitored, using change detection as an early indicator of infection, malignancy, burn victims, a non-healing wound, and dry skin conditions.

## Figures and Tables

**Table 1:** An overview of the descriptive stats of the human skin reflectance over a sample of 50 healthy participants at a center frequency of 90 GHz.

Location	Mean	SD	SEM
The back of the hand	0.631	0.093	0.013
The palm of the hand	0.563	0.097	0.014
Volar side of forearm	0.633	0.066	0.009
Dorsal surface of forearm	0.565	0.073	0.010
Inner wrist	0.680	0.065	0.009
Outer wrist	0.618	0.065	0.009
All locations	0.615	0.088	0.012

**Table 2:** A comparison in the mean reflectance values of the skin for males and females over all locations.

Location	Mean Female	SD Female	Mean Male	SD Male
The back of the hand	0.641	0.085	0.624	0.097
The palm of the hand	0.579	0.097	0.553	0.096
Volar side of forearm	0.641	0.055	0.628	0.072
Dorsal surface of forearm	0.573	0.066	0.560	0.078
Inner wrist	0.704	0.051	0.664	0.068
Outer wrist	0.634	0.064	0.607	0.064
All locations	0.629	0.085	0.606	0.089

**Table 3:** Significance level of the mean differences in the reflectance values between both sexes groups.

Location	Mean Difference in Reflectance Between Male & Female Groups	Independent T-Test Uncorrected *p*-Value	Significance Level
The back of the hand	0.017	0.518	Not Significant
The palm of the hand	0.026	0.351	Not Significant
Volar side of forearm	0.013	0.452	Not Significant
Dorsal surface	0.013	0.553	Not Significant
Inner wrist	0.04	0.025	Yes Significant
Outer wrist	0.027	0.159	Not Significant

**Table 4:** Significance level of the mean differences in the reflectance values between all measurements locations of females’ sample.

Locations	Mean Difference in Reflectance for the Two Measurement Locations	Paired *T*-Test Uncorrected *p*-Value Females’ Sample	Significance Level
Back-palm	0.0620	1.0 × 10^−6^	Yes Significant
Back-dorsal	0.0690	3.2 × 10^−4^	Yes Significant
Back-volar	0.0002	0.9930	Not Significant
Back-inner wrist	0.0630	1.9 × 10^−3^	Yes Significant
Back-outer wrist	0.0073	0.6260	Not Significant
Palm-dorsal	0.0069	0.7290	Not Significant
Palm-volar	0.0621	0.0070	Yes Significant
Palm-inner wrist	0.1245	1.1 × 10^−5^	Yes Significant
Palm-outer wrist	0.0546	4.8 × 10^−2^	Yes Significant
Dorsal-inner wrist	0.1314	1.8 × 10^−9^	Yes Significant
Dorsal-outer wrist	0.0615	5.7 × 10^−5^	Yes Significant
Volar-dorsal	0.0690	4.0 × 10^−6^	Yes Significant
Volar-inner wrist	0.0625	2.9 × 10^−7^	Yes Significant
Volar-outer wrist	0.0075	0.4330	Not Significant
Inner-outer wrist	0.0700	3.1 ×10^−7^	Yes Significant

**Table 5:** Significance level of the mean differences in the reflectance values between all measurements locations of males’ sample.

Locations	Mean Difference in Reflectance for the Two Measurement Locations	Paired *T*-Test Uncorrected *p*-Value Males’ Sample	Significance Level
Back-palm	0.0710	1.6 × 10^−10^	Yes Significant
Back-dorsal	0.0640	4.0 × 10^−6^	Yes Significant
Back-volar	0.0034	0.7763	Not Significant
Back-inner wrist	0.0399	2.6 × 10^−3^	Yes Significant
Back-outer wrist	0.0171	0.1152	Not Significant
Palm-dorsal	0.0076	0.5609	Not Significant
Palm-volar	0.0751	2.0 × 10^−6^	Yes Significant
Palm-inner wrist	0.1116	6.3 × 10^−11^	Yes Significant
Palm-outer wrist	0.0546	1.4 × 10^−5^	Yes Significant
Dorsal-inner wrist	0.1040	3.8 × 10^−10^	Yes Significant
Dorsal-outer wrist	0.0469	1.3 × 10^−4^	Yes Significant
Volar-dorsal	0.0680	1.2 × 10^−11^	Yes Significant
Volar-inner wrist	0.0365	4.7 × 10^−4^	Yes Significant
Volar-outer wrist	0.0205	3.2 × 10^−2^	Yes Significant
Inner-outer wrist	0.0570	1.2 × 10^−9^	Yes Significant
